# Amniotic fluid C-reactive protein as a predictor of infection in caesarean section: a feasibility study

**DOI:** 10.1038/s41598-018-24569-8

**Published:** 2018-04-23

**Authors:** Zbigniew Marchocki, Angela Vinturache, Kevin Collins, Paddy O’ Reilly, Keelin O’Donoghue

**Affiliations:** 1 Department of Obstetrics and Gynaecology, University College Cork, Cork University Maternity Hospital, Cork, Ireland; 2 John Radliffe Hospital, Oxford University Hospitals NHS Foundation Trust, Oxford, United Kingdom; 3 Department of Microbiology, University College Cork, Cork, Ireland; 4 The Irish Centre for Fetal and Neonatal Translational Research (INFANT), University College Cork, Cork, Ireland

## Abstract

This study evaluated the feasibility of maternal C-reactive protein (CRP) in amniotic fluid (AF) as a predictor of post-partum infection in women who undergo emergency or elective caesarean section (CS). AF bacterial culture and levels of hs-CRP in maternal serum and AF were evaluated in Day 0 and three days thereafter (Day 3) in 79 women undergoing CS. Univariate analyses assessed the clinical and demographic characteristics, whereas the ROC curves assessed the feasibility of hs-CRP as marker of inflammation in women who undergo CS. There was no difference in AF, Day 0, and Day 3 serum hs-CRP levels between women with sterile compared to those with bacterial growth in AF. Among women with positive AF cultures, AF and Day 0 serum hs-CRP levels were higher in women who underwent emergency compared to those who had elective CS (p = 0.04, and p = 0.02 respectively). hs-CRP in Day 0 and Day 3 serum but not in AF has a fair predictor value of infection in emergency CS only (AUC 0.767; 95% CI 0.606–0.928, and AUC 0.791; 95% CI 0.645–0.036, respectively). We conclude that AF hs-CRP is not feasible in assessing the risk of post-cesarean inflammation or infection.

## Introduction

The proportion of births delivered by caesarean section (CS) has increased worldwide significantly over the past three decades, currently approximately 18.6% of pregnancies being delivered by CS^1^. There is considerable variation among countries and regions, particularly in rates of emergency CS, the reported rates of CS ranging from 6.0% to 27.2% in the least and most developed regions, respectively^2^. For instance, CS rates vary from 1% in Nigeria to 46% in Brazil^3^. High CS rates have been reported in the UK and Australia, where 26.5% and 32.3% of births are by CS, respectively^4^. In the USA, was reported a 41% increase in incidence in a 13-year period^5^, while in Ireland the CS rate increased from 20.5% in 1999 to 25.6% in 2016^6,7^. As CS has become safer and electively scheduled, functional and cosmetic aspects, as well as risks of hospital-acquired infection have gained increased importance^8^.

Women undergoing CS have a 5 to 20-fold greater risk for infectious morbidity compared to vaginal delivery^9^. It is estimated that postoperative rates of infection after CS range between 1.2 and 5.0% for women during their inpatient stay^8,10–12^. It is likely that the overall rates of infection may be even higher than these estimates considering the typical short postoperative hospital stay of 2 to 4 days after a CS. Wound infection, urinary tract infection, endometritis and fever occur in 8% of women after CS^13^. Most frequent infectious complications following cesarean birth include fever (febrile morbidity), skin and soft tissue infection (wound infection), endometritis (inflammation of the lining of the uterus), and urinary tract infection which occur in 8% of women. Occasionally, serious infectious complications may occur including pelvic abscess, bacteremia, septic shock, necrotizing fasciitis, and septic pelvic vein thrombophlebitis which can lead to maternal mortality^14,15^. It is estimated that around 80% of complications occur after hospital discharge. With the increase in CS rates worldwide, it is anticipated that related infections will become an increasing health and economic burden^1,13^, through their associated morbidity, increased hospital stay or re-admission after delivery^13^.

Factors associated with an increased risk of infection in women after CS include emergency procedures, duration of labor, ruptured membranes, vaginal examination in labor, internal fetal monitoring, obesity, blood loss and operative technique^1,13^. The most important source is the genital tract, especially if membranes are ruptured before delivery. Even during normal delivery, 70–90% of amniotic fluid becomes colonized with microbes^16^. Given the importance of these infections, and the absence of effective surveillance following CS, it is imperative to develop methods able to predict the risk of postoperative infection after CS that could be implemented at admission for labour and delivery.

C-reactive protein (CRP), an acute phase reactant protein, is a biological marker of value in detecting infectious complications. CRP is a member of pentraxin family of proteins, that is secreted into the circulation from the liver as a reaction to the onset of inflammation and/or acute tissue injury. CRP is also produced by cells in the vascular wall such as endothelial cells, smooth muscle cells, and also by the adipose tissue. CRP has been extensively examined in the context of preterm prelabour rupture of membranes premature labor and delivery^17–19^. Maternal serum CRP was assumed to be a clinical indicator for women with preterm labour or preterm rupture of membranes related to the possible preceding subclinical maternal infection^18,20^. Maternal CRP concentration level was shown to be significantly higher before, during, and after the labour in patients with Fetal Inflammatory Response Syndrome (FIRS)^21^. However, there have been no recent studies performed on the use of amniotic fluid (AF) markers as predictors of postoperative infectious morbidity post CS in term pregnancies.

Whereas standard CRP tests traditionally measure CRP down to concentrations of 3–5 mg/L, high sensitivity CRP (hs-CRP) also called ultra-sensitive CRP, measures down to levels around 0.3 mg/L. This improved sensitivity allows hs-CRP to be used to detect low-grade inflammation (<2.0 mg/L). Since higher values (>10 mg/l) confirm clinically evident infection, lower values might suggest chronic inflammatory processes, subclinical infection, or early stage infection. Therefore, elevated hs-CRP in AF could suggest an evolving infectious process and might have a role in predicting the risk of postoperative infectious morbidity.

Isolating bacterial organisms from the AF would provide a final diagnosis of infection, being considered the gold standard in diagnosis of infection. However, this is time-consuming and often is negative, since up to 99% or more of microbial species visualized by microscopy are uncultivable. Thus, measurement of AF hs-CRP might have a role in diagnosing subclinical or an early infectious process that has already started in utero, which might not manifest as an active infection until later in the post-partum period.

This study was designed to evaluate the feasibility of hs-CRP measurement in AF at the time of CS in predicting inflammation in early postpartum period. In addition, by comparison, we assessed whether hs-CRP levels in serum at the time of CS and in the immediate postpartum period is correlated with postnatal infectious complications.

## Methods

### Ethical approval

The study was approved by the Clinical Research Ethics Committee of the Cork University Hospital (ECM 4: 02/08/2011). Informed written consent was obtained from the study participants at the time of recruitment, for use of clinical information and biological sample collection. All experiments were performed in accordance with the relevant guidelines and regulations.

### Study population

This was a prospective observational cohort study of 79 pregnant women undergoing CS at Cork University Hospital, a large teaching hospital, with around 8,500 deliveries per year in Cork, Ireland. Women were recruited to participate in the study at admission for labour and delivery. Inclusion criteria were: gestational age ≥37 + 0 weeks, singleton pregnancies, maternal age ≥18 years. Women with current antibiotic use, premature rupture of membranes, preterm delivery, multiple pregnancy, active inflammatory and rheumatologic medical conditions, infectious disease, or other chronic condition that could potentially introduce bias were excluded from the study. Gestational age at delivery was determined based on the first trimester ultrasound scan. All women were offered a routine dating scan at the first prenatal visit to confirm gestational age and to accurately determine the estimated date of confinement, as part of the standard of practice.

### Study design

Participants were divided into two groups: women who underwent elective caesarean section (n = 35) and women who had an emergency caesarean section (n = 44). Maternal blood and amniotic fluid were collected from all participants. Samples obtained were coded to maintain confidentiality and to eliminate operator bias.

Maternal blood was obtained via venipuncture of the cubital vein. Biological samples were collected at admission, before the CS was performed, and three days postoperatively. Serum samples were collected using EDTA blood collection tubes and stored at −80 °C until analysis. The samples had not undergone more than two thaw–freeze cycles before CRP levels were measured.

Maternal serum hs-CRP levels were determined by enzyme-linked immunosorbent assay (ELISA) using a commercially available electrochemiluminescent multiplex assays (Meso Scale Discovery 96-Well Multi-Array CRP Assay, Meso Scale Diagnostics, MD, USA). The assay was performed according to manufacturer instructions. Each sample was examined in duplicate. The method had a sensitivity of 0.33 pg/L and a range of detection of 1.33–49 608 pg/mL, being able to measure analytes in very low sample volumes, as it was our case, and without multiple dilutions^22^.

Amniotic fluid specimens were obtained via direct syringe aspiration at the time of the CS and stored at 4 °C until analyzed. AF samples were processed for hs-CRP, bacterial count, and culture. AF hs-CRP levels were measured using the same assay used for analysis of serum samples (Meso Scale Discovery (MSD) 96-Well Multi-Array CRP Assay, Meso Scale Diagnostics, MD, USA), following the manufacturer protocol. AF samples were plated on several media for bacterial identification. Colombia Blood Agar, and subsequent Gram stain, catalase test, Baird-Parker agar base plates, and Rapid Agglutination Test were employed for the identification of bacterial species.

### Maternal and neonatal variables

Demographic and clinical variables were abstracted from the clinical records for admission to labour and delivery. Information about pregnancy, labour and delivery was linked to information on newborn infant using unique identifiers (hospital numbers). The information was collected for four categories of variables: (i) maternal variables, which included maternal age, BMI, ethnicity, smoking, use of recreational drugs, parity, gestational age at delivery, pregnancy complications, antibiotic use during pregnancy; (ii) infant variables included birth weight, colour of liquor, Apgar score at 1 min, gender, cord pH measurements; (iii) indication for CS (failure to advance or fetal distress), stage of labor when the CS was performed; and (iv) characteristics of labor, including onset of labor, stage of labor, rupture of membranes, number of vaginal examinations, interventions in labor, and use of antibiotics in labor.

### Study outcomes

The main outcome of the study was presence of an infectious process associated with CS, which was defined as a composite variable of clinical symptoms (pyrexia in labour), diagnosis of postnatal infection (endometritis, wound infection), bacterial growth, and use of antibiotics in labour. Secondary outcomes were neonatal complications including fetal wellbeing at birth, infection, and neonatal unit admission. Apgar score was used as a proxy to assess fetal wellbeing at birth.

### Statistical analysis

Patient data was anonymized, coded, and entered into an Excel datasheet. Descriptive statistics were used to present the demographic and clinical characteristics of the two groups of patients, emergency and elective CS. Categorical variables were compared using the Chi-square test and presented as percentage (%). Continuous variables were analysed by one-way analysis of variance ANOVA, or Student t-test, as appropriate. The non-normally distributed data of the hs-CRP assays were logarithmically transformed. Pearson’s product-moment coefficient was used to measure the dependence between the hs-CRP levels in AF and serum in elective and emergency CS, in women with and without bacterial growth. The area under the curve (AUC) obtained by the Receiver Operator Curve (ROC) assessed the ability of hs-CRP measurement in AF and Day 0 and Day 3 serum in predicting CS-associated infection/inflammation in women who had either emergency or elective CS.

All reported *p*-values are 2-sided, and *p*-values < 0.05 were considered statistically significant. Data were analyzed using Microsoft Statistical Package for Social Sciences for Windows v23 (SPSS Inc, Chicago, Illinois).

## Results

### Characteristics of the study population

From the 79 women undergoing CS recruited in the study, five were excluded from the final analysis due to inadequate AF samples. The demographic and clinical characteristics of the 74 subjects included in the study are shown in Table 1. The average age of women at the time of enrollment was 32.7, ranging from 20 and 42 years old. The majority of women were of Irish descent (87.8%), did not smoke during pregnancy (82.4%), had a BMI higher than 25 kg/m^2^ (61.8%), and delivered after 39 weeks gestation (71.7%). The distribution of the gestational age at delivery was as follow: 4 women (5.4%) delivered at 37 weeks, 17 (23.0%) at 38 weeks, 23 (39.1%) at 39 weeks, 9 (12.2%) at 40 weeks, and 21 (28.4%) women after 41 weeks gestation. Almost half of women (45.9%) were nulliparous and approximately a third had a history of miscarriage (25.7%). Most women were in good health, with no preexisting medical conditions (60.8%), and had no pregnancy complications during the index pregnancy (63.5%).

**Table 1:** Socio-demographic and clinical characteristics of the study population.

Characteristics	All women (n = 74)^a^	Elective CS (n = 35)	Emergency CS (n = 39)	p-value
Age (mean ± SD) (range) (years)	32.7 ± 4.8 (20–42)	34.7 ± 4.1 (24–42)	30.9 ± 4.7 (20–41)	0.680
Parity				
Nulliparous	34 (45.9)	6 (17.6)	28 (82.4)	<0.001*
Multiparous	40 (54.1)	29 (72.5)	11 (27.5)	
Nationality n (%)				
Irish	65 (87.8)	31 (89)	34 (87)	0.250
Non-Irish	9 (12.2)	4 (11)	5 (13)	
BMI (kg/m^2^) n (%)				
18.0–24.9 kg/m^2^	26 (38.2)	11 (42.3)	15 (57.7)	0.507
>25.0 kg/m^2^	42 (61.8)	11 (45.2)	23 (54.8)	
Smoking in pregnancy	13 (17.6)	6 (17.1)	7 (17.9)	0.920
Preexisting medical conditions ^b^	29 (39.2)	10 (34.5)	19 (65.5)	0.620
Miscarriage n (%)				
Nil	55 (74.3)	24 (69)	31 (79)	0.120
One or more	19 (25.7)	11 (31)	8 (21)	
Gestational age at delivery (weeks) median (range)	39 (37–42)	39 (37–40)	41 (37–42)	0.001*
Pregnancy complications^c^ n (%)				
No	47 (63.5)	24 (68.5)	23 (58.9)	0.390
Yes	27 (36.5)	11 (31.5)	16 (41.1)	

^a^May not add to the total number due to missing.

^b^Preexisting medical conditions include: asthma, hypothyroidism, irritable bowel syndrome, Chron’s disease, depression, and anxiety.

^c^Complications included: intrauterine growth restriction, antepartum haemorrhage, threatened preterm labour, reduced amniotic fluid index, urinary tract infection, Group B Spreptococcus positive, Hyperemesis Gravidarum.

BMI, Body mass index.

Of the 74 participants, 47% (35/74) of women underwent elective CS and 53% (39/74) emergency CS. As expected, the two groups differed by parity and gestational age (*p* < 0.001 for both) (Table 1). This was likely secondary to the fact that most of the elective CS were repeat procedures at 39 weeks gestation. However, the elective and emergency group did not differ significantly by BMI, nationality, smoking, or pregnancy complications (Table 1).

Characteristics of labour, delivery, and postpartum infectious morbidity in the two groups, elective and emergency CS, are presented in Table 2. The primary indication for elective CS was repeat elective CS (83%), whereas presumed fetal compromise with non-reassuring CTG was the most frequent indication for emergency CS (54%). Women who underwent emergency CS were more likely to have had their labour induced (71.8%) (p < 0.001). Among induction methods, 46.2% of women who delivered by emergency CS were administrated PGE2. Ninety seven percent of women who had an elective CS had intact membranes, whereas in the emergency CS group, 92% had membranes ruptured either spontaneously or artificially. Six women in the emergency CS group developed pyrexia in labour and ten received antibiotics. Infectious morbidity in postpartum was present in 17% of women who had elective CS and 8% of women who underwent emergency CS.

**Table 2:** Characteristics of labor, delivery, and postpartum infectious morbidity.

Characteristic	Elective CS (n=35)	Emergency CS (n=39)
**Indication for CS n** (**%)**		
Elective CS		
Repeat CS	29 (83)	—
Breech presentation	3 (9)	—
Other indication	3 (9)	—
Emergency CS		
NRCTG	—	21 (54)
FTP	—	9 (23)
Failed IOL	—	7 (18)
Malpresentation	—	1 (3)
Other	—	1 (3)
**Cervical dilation n (%)**		
1–3 cm	—	20 (53)
4–6 cm	—	8 (21)
7–9 cm	—	6 (16)
10 cm	—	4 (11)
**Antibiotics at CS n (%)**		
Given	34 (97)	39 (100)
Not given	1 (1)	—
**Onset of labor n (%)**		
Not in labor	34 (97.1)	1(2.7)
SOL	1 (2.9)	9 (23.1)
IOL	—	28 (71.8)
**Mode of labour induction n (%)**		
PGE2	—	18 (46.2)
ARM	—	2 (5.1)
Syntocinon	—	2 (5.1)
No induction	—	17 (43.6)
**Length of the 1** ^**st**^ **stage** ^**1**^ **(h)**	—	14.00 (4–45)
**Length of the 2** ^**st**^ **stage** ^**1**^ **(h)**	—	1.00 (1–2)
**Membranes at the time of CS n (%)**		
Intact	34 (97.1)	3 (8)
SROM	1 (2.9)	14 (36)
ARM	—	22 (56)
Interval ROM to delivery^1^ (h)	2h only in 1 case	10.30 (1–59)
**Number of VE**		
Before ROM^1^	—	3.00 (0–7)
After ROM^1^	—	6.00 (0–13)
**Number of FBS in labor n(%)**		
Nil	—	26 (67)
1	—	10 (27)
2	—	2 (5)
3	—	1 (3)
**Pyrexia in labor n (%)**		
Absent	—	33 (85)
Present	—	6 (15)
**Antibiotics in labor n (%)**		
No	—	29 (74)
Yes	—	10 (26)
**Cord pH n (%)**		
Not performed	—	26 (67)
Arterial		
>7.25	—	3 (8)
7.20–7.25	—	4 (10)
<7.20	—	5 (13)
Venous		
>7.25	—	9 (23)
7.20–7.25	—	1 (3)
<7.20	—	3 (8)
**Postpartum infection n (%)**		
Absent	29 (83)	36 (92)
Present	6 (17)	3 (8)
Wound infection	2 (6)	3 (8)
Endometritis	3 (9)	—
Pyrexia	1 (3)	—
**Newborn Outcomes**		
Apgar score 1 min >7	34 (100.0)	36 (94.7)
Apgar score 5 min >7	34 (100.0)	38 (100.0)
Neonatal unit admission	2 (5.7)	4 (10.3)
Birth weight		
<2,500g	1 (1.4)	1 (1.4)
2500–4000g	29 (39.2)	28 (37.8)
>4000g	5 (6.8)	10 (13.5)
Gender		
Female	16 (21.6)	21 (28.4)
Male	19 (25.7)	18 (24.3)

Abbreviations: NRCTG, non-reassuring CTG; FTP, failure to progress; IOL, induction of labour; SOL, spontaneous onset of labour; PGE2, prostaglandin E2; ARM, artificial rupture of membranes; SROM, spontaneous rupture of membranes; ROM, rupture of membranes; FBS, fetal blood sampling.

^1^Data presented as median (range). Note: The length of the 1^st^ stage includes latent, active, and transitional phase.

Secondary neonatal outcomes are presented in Table 2. More than 97% (70/74) of babies had an Apgar score higher than 7 at one minute, and all babies had Apgar score higher than 7 at 5 minutes. Eight percent of newborns were admitted to neonatal intensive unit (6/74) for congenital pneumonia (n = 1), transient tachypnea of newborn (n = 3), or for other causes (n = 2). Two newborns had a birth weight less than 2500 g, and 15 babies were macrosomic, weighting more than 4000 g, with the rest being adequate for gestational age (birthweight 2500–4000 g). The gender distribution was equivalent (50% female and 50% males) (Table 2).

### Feasibility of AF sample collection

In 6% of cases (5/79), AF could not be analyzed after CS, as it was either of insufficient amount or could not be processed due to thick meconium. Adequate AF sample were obtained from all the elective CS. In the emergency CS group, 26% of samples (10/39) were obtained through intact amnion, while the rest of samples were obtained after amnion incision or rupture. In 7% of the emergency cases (3/44) there was little amniotic fluid left around the baby. In 5% of emergency cases (2/44) the operator was able to aspirate thick meconium; however, due to its consistency, preparation of the sample for the hs-CRP assay measurement (separation by centrifugation) was not possible. In the elective group, 86% of amniotic fluid samples (30/35) were obtained by direct needle aspiration through intact amnion. The remaining 14% (5/35) of samples were obtained after unintended spontaneous rupture of the amnion.

### Bacterial growth in AF samples

Bacterial count was performed for all 74 women from whom an amniotic fluid sample was obtained. Almost 60% (44/74) of AF samples collected showed bacterial colonization: 46% of elective CS (16/35) and 72% of emergency CS cases (28/39). Among the AF samples with positive bacterial growth, 43% (19/44) had subsequent bacterial typing performed. Every bacterial isolate was Gram stained and catalase tested. Further testing included antibiotic disc sensitivity testing, MRSA, and Baird-Parker selection agar testing and agglutination tests. From these presumptive tests, five isolates were selected for confirmatory StaphAPi analysis, as all presumptive tests had indicated towards *Staphylococcus*. Three showed *Staphylococcus* epidermidis and the other two showed *Staphylococcus* lugdunensis. That was an interesting discovery as *Staphylococcus* lugdunensis, a virulent coagulase-negative staphylococcus, has not been previously isolated from amniotic fluid^22^. Bacterial growth was almost 3 times more likely to occur in emergency CS as compared to elective CS (OR 3.0, 95% CI 1.2–7.9).

### hs-CRP levels in AF and serum of women undergoing CS

hs-CRP was measured in AF samples collected from 74 women, 35 who underwent elective and 39 who had emergency CS (100% of sample). All 35 women in the elective group and 38 of women in the emergency group (97%, 38/39) had a pre-operative blood sample collected for measurement of hs-CRP (Day 0 hs-CRP). More than 90% of women provided a blood sample in the day 3 postoperatively for measurement of hs-CRP (Day 3 hs-CRP), 33 women in the elective group (94%, 33/35) and 35 women in the emergency CS group (90%, 35/39). Blood samples were not collected from six women, who were discharged earlier than day 3 postoperatively from the hospital.

Mean AF hs-CRP levels were approximately 10 times higher among the women undergoing emergency CS (1,333 ng/ml) compared with women who had an elective CS (134 ng/ml; p = 0.009) (Table 3).

**Table 3:** hs-CRP levels in AF and maternal serum measured at elective and emergency CS.

hs-CRP levels (10^3^ ng/ml)	Elective CS n = 35	Emergency CS n = 39	*p-value*
Amniotic fluid	0.134 (0.050–0.218)	1.333 (0.394–2.272)	0.004*
Day 0 serum	7.965 (4.009–11.923)	134.6 (95.868–365.165)	<0.001*
Day 3 serum	97.805 (72.884–122.726)	75.892 (54.636–97.147)	0.959

Data are presented as mean (95% CI).

Student *t-test* applied to compare between elective and emergency CS hs-CRP in logarithmically transformed data.

hs-CRP levels were higher in the serum collected in Day 0 in women who underwent emergency CS than in women who had planned CS (p < 0.001) However, at day three (Day 3 serum) the levels of hs-CRP did not vary significantly between the elective (mean 97,805 ng/ml) and emergency group (mean 75,892 ng/ml) (p = 0.959) (Table 3).

### Correlation between clinical and demographic characteristics and hs-CRP levels in women who underwent emergency or elective CS

Among women with bacterial growth in the AF samples, levels of hs-CRP were higher in the AF (p = 0.041) and Day 0 serum (p = 0.020) but not in Day 3 serum (p = 0.070) in women who underwent emergency CS compared to women who had an elective CS.

Among women who developed postpartum infection, hs-CRP was higher in Day 3 serum in women who underwent elective CS than in women with emergency CS (p = 0.021), whereas there was no difference in hs-CRP levels in Day 0 serum or AF.

There was a significant difference in hs-CRP levels in AF and Day 3 serum samples of women with a BMI > 25 kg/m^2^, hs-CRP being higher in AF and lower in Day 0 serum of women with increased BMI (overweight and obese) who underwent emergency CS (p = 0.007 and p = 0.003, respectively) compared to women who had an elective CS. Women who smoked during pregnancy from the elective CS group had significantly higher Day 0 serum hs-CRP levels than women from emergency CS group (p = 0.038). There was no difference in the hs-CRP levels in the serum or AF of smokers who underwent emergency CS or in Day 3 serum and AF of smokers who had an elective CS.

In the emergency CS group, the levels of hs-CRP were higher in Day 0 serum in women who had at least one vaginal examination in labour (p < 0.001) as compared to women who had no examination, and Day 3 serum in women who had pyrexia in labour (p = 0.003) as compared to women who had normal body temperature. No other clinical and demographic risk factors (parity, number of miscarriages, preexistent medical conditions, nationality, antibiotics administered in labour, fetal blood sampling, or membrane rupture) in the emergency CS group had a significant influence on serum or AF hs-CRP levels.

Parity influenced the levels of hs-CRP in elective CS group. In this group, multiparous women had higher levels of hs-CRP in Day 3 serum than nulliparous women (p < 0.001).

A Pearson correlation was run to determine the relationship between the levels of hs-CRP in AF and serum in women who underwent CS. Overall, there was a moderate, positive correlation between hs-CRP levels in AF and Day 0 serum (r = 0.453, p < 0.001) and a weak correlation between hs-CRP levels in AF with Day 3 serum (r = 0.270, p = 0.031). When stratified by the type of CS, in women who underwent emergency CS, a positive correlation was noted between hs-CRP levels in AF with Day 0 serum (r = 0.443, p = 0.006), but not with Day 3 serum (r = 0.267, p = 0.127). Statistically significant correlations were observed between hs-CRP from Day 0 serum with hs-CRP levels in the AF (r = 0.754, p < 0.001) and with Day 3 levels of hs-CRP in serum (r = 0.467, p = 0.007) in women who underwent elective CS. The odds of evidence of an inflammatory process were 3.6 times higher in emergency CS than in elective CS (OR 3.6, 95% CI 1.3–10.1) (Supplemental Figure 1).

The area under the curve (AUC) obtained by the Receiver Operator Curve (ROC) assessed the ability of hs-CRP measurement in AF and serum in predicting inflammatory/infectious process in women who underwent either emergency or elective CS (Table 4). Whereas AUC was significant for hs-CRP measurement in Day 0 and Day 3 serum in emergency CS, indicating good value of hs-CRP in predicting inflammatory processes in these cases, the AUC showed low predicting value of the inflammatory marker in AF or serum in women who underwent elective CS (Table 4).

**Table 4:** Performance of hs-CRP in AF and serum for prediction of an inflammatory/infection process in women who undergo CS.

	All cases		Emergency CS		Elective CS	
	AUC	95% CI	AUC	95% CI	AUC	95% CI
hs-CRP in AF	0.590	0.457–0.722	0.558	0.331–0.784	0.518	0.316–0.721
hs-CRP in Day 0 serum	0.648	0.522–0.773*	0.767	0.606–0.928*	0.503	0.305–0.701
hs-CRP in Day 3 serum	0.605	0.460–0.749	0.791	0.645–0.036*	0.602	0.401–0.803

^*^p < 0.05.

## Discussion

This study evaluated the propensity of AF hs-CRP in predicting CS-associated infection and inflammation. By using ROC plot analysis, our findings suggest that AF hs-CRP does not predict inflammation in women who deliver by CS, whereas serum hs-CRP appears to be a better predictor of inflammation/infection in emergency than in elective CS. Considering low predictor value of infection and technical difficulties in harvesting and adequate sampling, AF hs-CRP does not appear a feasible marker to assess the risk of an infectious process in women who deliver by CS. However, hs-CRP may have value in identifying low levels of inflammation that may progress towards sepsis in labour or postnatal infection.

The choice of measuring hs-CRP in our samples was two-fold. Firstly, evidence shows that particular proteins such as CRP or other molecules could provide a “signature” for detection of “sublinical” amniotic fluid inflammation that may be predictive and could pick up infection at an early stage^23,24^. Secondly, we were interested to detect low levels of inflammation in small biologic samples. The commercially available hs-CRP assays use low amounts of sample (microliters). Nonetheless, previous studies have shown that, despite difference in the levels of detection, there is a strong, significant correlation between the levels of wide-range measured through standard immunoturbidometric assay (i.e. COBAS® Integra, Siemens Advia systems) and high-sensitivity CRP in biologic samples^25,26^. Furthermore, we were aware of potential sampling issues that could arise at the harvesting of AF, especially in women with ruptured membranes.

This study has shown the ability of measuring hs-CRP in less than 1 mL of AF in both elective and emergency CS deliveries. However, even though less than 1 mL of fluid was required for hs-CRP analysis, in three emergency cases no amniotic fluid was obtained. Also, the consistency of the amniotic fluid sample influenced the chance of success. In two of the emergency CS cases thick meconium aspirated with a plain syringe proved the sample preparation inadequate for the hs-CRP assay, although bacterial colonization could still be analyzed. Consequently, we conclude that analysis of AF hs-CRP can be limited at the CS by both, insufficient amount or inadequate consistency of AF samples.

Contamination of the amniotic fluid samples with maternal blood and cells may have posed pre-analytical bias to our study and should also be taken into consideration, particularly for the cases of pre-ruptured amnion^27^. Although for most of the samples in elective CS group the AF samples were harvested through the intact amnion, the pre-contamination with maternal cells was still possible. Thus, it is possible that the levels of hs-CRP may not truly reflect the levels of this inflammatory marker in the AF. Routinely harvesting AF samples in emergency CS may not be always feasible. The operator may be reluctant to do so when trying to deliver a presumed compromised fetus without delay^28^. Furthermore, the risk of fetal skin damage by puncture associated with the use of needle aspiration described in other studies needs to be appreciated, although these risks did not materialize in our study.

Despite measures to decrease the incidence of postoperative infection, such as changes in operative techniques and prophylactic antibiotics before skin incision^1^, the rate of infectious morbidity after CS remains high. Therefore, finding a new method or marker that could predict the risk of sepsis and postoperative infection would be extremely useful. In our study, AF hs-CRP levels were significantly higher in emergency compared to elective CS cases, but whether this can be used clinically and to what benefit is still uncertain. It is conceivable that AF hs-CRP measurement might be of more relevance in those emergency cases that occur at the end of a prolonged labor, where the risk of post-partum infectious morbidity is much higher.

The higher levels of Day 0 serum hs-CRP as compared to Day 3 in women who delivered by emergency C-section may reflect increase in the inflammatory marker following rupture of membranes and physiological changes in response to elevated serum concentrations of estrogen, progestogen and prostaglandins^20,29^ or to the tissue injury consecutive the procees of labour itself^29^. The rise in Day 0 CRP levels might have been mediated, at least in part, by the stress response associated to surgical trauma and additional potentiating effects of anesthesia^30^.

In contrast, Day 3 serum hs-CRP levels were significantly elevated in the elective CS group in comparison to the pre-operative level. For these cases it is likely that the higher postoperative levels may be a consequence, at least in part, to tissue trauma associated with the operation. Similar with our study, a previous report from Keski-Nisula *et al*. showed a significant peak of serum CRP on day 2 In parturients operated upon after the onset of labor or ruptured membranes^18,31^. The postoperative values remaining elevated up to the sixth postoperative day than in parturients operated upon with intact membranes or not in labor^31^. Several systematic reveiwes and meta-analyses assessed recently the predictive value of CRP in major abdominal surgery, showing that day 3 postoperative CRP levels are predictive of infectious complications and may aid patient selection for safe and early hospital discharge, guide the need for additional investigations, or prevent overuse of imaging^32,33^.

Intra-amniotic inflammation is a risk factor for adverse pregnancy and neonatal outcome, regardless of the presence or absence of a positive amniotic fluid culture^34^. It has been shown that bacteria have the ability to cross through intact membranes^35^ and asymptomatic microbial colonization has been reported as a cause of preterm premature rupture of membranes and pre-term labor. This could explain the positive amniotic fluid cultures in 46% of women in our elective group; the number could be much higher since up to 99% or more of microbial species visualized by microscopy are uncultivable^36^. This would strengthen the potential role of AF hs-CRP in diagnosing subclinical or an early infectious process that has already started in utero. Since hs-CRP does not cross the placenta values obtained from the AF represent fetal source as a reaction to external or internal stimuli^37^.

The main limitation of our study is the relatively small sample size. However, our primary aim was to examine the feasibility of measurement of hs-CRP in AF and not to definitely prove its association with post-partum infectious morbidity. Furthermore, we did not include in our analyses the duration of labour prior to surgery, with or without ruptured membranes, the duration of the operation itself, or the duration of internal monitoring before operation.

We appreciate that information on endometrial, placental, and membrane histology to determine whether inflammatory reactions were present in cases with raised hs-CRP would have been instrumental in differentiation between inflammation and infection. Placental cultures, should they have been available to this study, would validate the AF culture findings.

We acknowledge that several new biomarkers of inflammation have been recently studied. Among many of the inflammatory markers potentially available, very few were found of value for routine clinical practice. For instance, procalcitonin, is a recently discovered, promising biomarker of inflammation. Its value has been tested in blood and seems to be more significant for sever sepsis/septic shock^38^. Although procalcitonin has proved to be a potential marker for sepsis, its physiological role remains still uncertain^39^. In addition, the normal values of procalcitonin in pregnancy have not been defined and validated and there is little information on its value in pregnancy^40^. Furthermore, although procalcitonin has been examined previously in blood and amniotic fluid, those studies have not been translated into clinical practice^41^. Thus, our preference to hs-CRP reside in the fact that this is a commoly used marker of inflammation in routine clinical practice, with values and clinical significance most clinicians are familiar. 

Erythorcyte sedimentation rate (ESR) is routinely used as a common marker of inflammation in clinical practice. We did not included ESR in our study for several reasons. Firstly, there is evidence in the literature of the association between CRP and not ESR with inflammation in pregnant and non-pregnant patients. CRP values des not vary with gestational age or other pregnancy complications. Secondly, ESR indirectly measures inflammation in the blood by assessing the rate at which red blood cells sediment in a period of one hour. Thirdly, ESR is a non-specific test, not reliable as a measure of inflammation in pregnancy. ESR is increased in several circumstances beside infection/inflammation including anaemia, malignant disease, aging, and pregnancy^42^. Previous studies have shown that, in pregnancy, ESR levels are dependent on gestational age, anaemia, low albumin, other pregnancy complications^43^. Caesarean section in itself may influence the value of ESR. Furthermore, by its definition, ESR is a blood test, which would preclude its use as a desired test of amniotic fluid. In addition, we did not add this test to our methodology to avoid bias due to high frequency of these two tests in providing discordant results^44^.

In conclusion, prediction of postpartum and post-operative infectious complications remains a great challenge after caesarean section. This study has proven the ability of measuring hs-CRP in less than 1 ml of AF in both elective and emergency CS settings. In our study, AF hs-CRP levels were significantly higher in emergency compared to elective CS, but whether or not this test can be used in a clinical setting warrants further investigation in larger studies. The wide range of serum CRP concentrations during the puerperium precludes, however, its use as a simple serum marker of obstetric postoperative complications.

## Electronic supplementary material

Supplementary Information
