# Supplementary material for: Amniotic fluid C-reactive protein as a predictor of infection in caesarean section: a feasibility study
Source: Sci Rep. 2018 Apr 23;8:6372. doi: 10.1038/s41598-018-24569-8 (PMC5913132; doi:10.1038/s41598-018-24569-8)
Supplement: Supplementary file 1 — Supplementary Information [file 41598_2018_24569_MOESM1_ESM.pdf]

MANUSCRIPT TITLE: Amniotic fluid C-reactive protein as a predictor of infection in caesarean section: a feasibility study

RUNNING TITLE: CRP in amniotic fluid

AUTHORS:

Zbigniew Marchocki,[a] Angela Vinturache,[b] Kevin Collins[c], Paddy O' Reilly,[c] Keelin O'Donoghue[a,d]

[a]Department of Obstetrics and Gynaecology, University College Cork, Cork University Maternity Hospital, Cork, Ireland

[b]John Radliffe Hospital, Oxford University Hospitals NHS Foundation Trust, Oxford, United Kingdom

[c]Department of Microbiology, University College Cork, Cork, Ireland

[d]The Irish Centre for Fetal and Neonatal Translational Research (INFANT), University College Cork, Cork, Ireland

Supplemental Figure 1. Correlation between hs-CRP levels in AF and serum in women who undergo CS

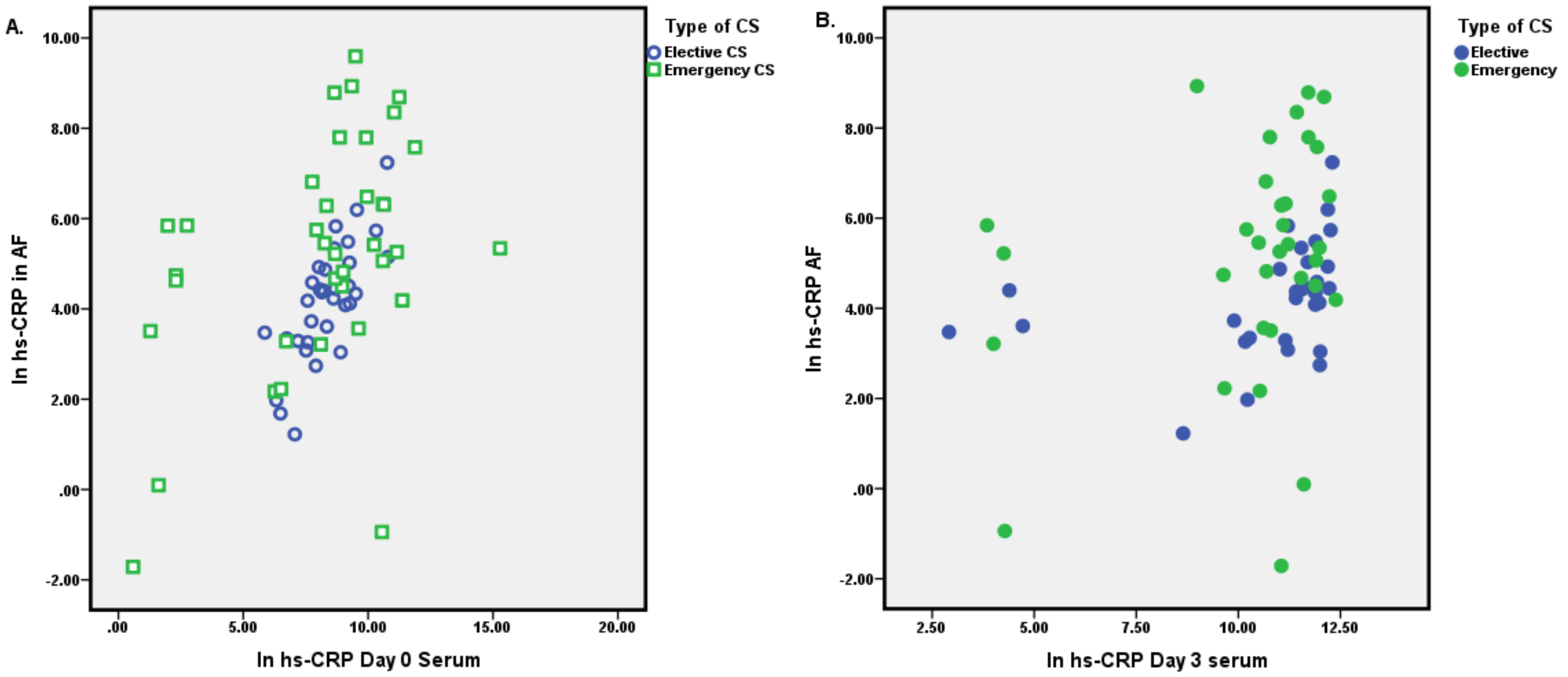

Data were logarithmically transformed.

Panel A shows a positive correlation between the levels of hs-CRP in AF and Day 0 in serum of women with elective CS and emergency CS, respectively. The correlation coefficients were r= 0.754, p<0.001 in elective CS and r=0.443, p=0.006 in emergency CS.

Panel B shows the relationships between the levels of hs-CRP in AF and Day 3 in serum of women with elective CS and emergency CS, respectively. The correlation coefficients were r=0.332, p=0.073 in elective CS and r=0.267, p=0.127 in emergency CS.
